# Reforming the cellular, molecular, and genetics course in a graduate biomedical science program: CIPP guided course evaluation

**DOI:** 10.12669/pjms.42.4.12712

**Published:** 2026-04

**Authors:** Fazal Arain, Satwat Hashmi, Kulsoom Ghias, Ambreen Surti, Rehana Rehman

**Affiliations:** 1 Fazal Arain, Department of Biological and Biomedical Sciences, Aga Khan University, Karachi, Pakistan; 2 Satwat Hashmi, Department of Biological and Biomedical Sciences, Aga Khan University, Karachi, Pakistan; 3 Kulsoom Ghias, Department of Biological and Biomedical Sciences, Aga Khan University, Karachi, Pakistan; 4 Ambreen Surti, Department of Biological and Biomedical Sciences, Aga Khan University, Karachi, Pakistan; 5 Rehana Rehman, Department of Biological and Biomedical Sciences, Aga Khan University, Karachi, Pakistan

**Keywords:** Biological &, Biomedical Sciences, CIPP, Curriculum reform, Graduate program, Program evaluation

## Abstract

**Background & Objective:**

Graduate programs in biological and biomedical sciences play a crucial role in developing competencies required for academic and research careers. Routine curricular evaluation of such programs is important to ensure their quality, impact and relevance. Cellular, Molecular and Genetic Sciences: Knowledge and Application (CMG) is one of the core courses of MPhil in Biological and Biomedical Sciences program. This study aimed to critically evaluate the CMG course, propose evidence-based reforms and compare student performance across student cohorts before and after the course reforms.

**Methodology:**

A CIPP(Context Input, Process, Product) guided, mixed-methods course evaluation (March 2024–June 2025) was conducted integrating document review, student feedback (scheduled mid- and end-of-module meetings and Virtual Learning Environment (VLE) based written evaluations), and faculty reflections with descriptive analysis of grade distributions across the 2023, 2024, and 2025 cohorts. Qualitative data were analyzed using directed content analysis mapped to CIPP domains, and quantitative data were summarized using descriptive statistics (counts, percentages; means ± SD where available). Findings were integrated through triangulation to inform iterative course refinements.

**Results:**

Data identified poor sequencing of content and insufficient formative assessments as the key issues in CMG course offered in 2023. In the first iteration (2024) CMG course was modularly restructured into three distinct segments: Cell Biology, Molecular Biology, and Genetics, supplemented with practice-based formative assessments. Data acquired after implementation of first iteration, recognized further simplification of genetic component in 2025. Periodic improvements in academic performance were observed; across 2023–2025, most students consistently achieved grades in the B (70–74) and C+ (65–69) categories, and relatively few students were found in the lower grade ranges. In 2025, there was a slight shift toward higher grades (A– and B+), with minimal failures or incomplete results across all three years.

**Conclusion:**

The feedback from students enabled systematic diagnosis and resolution of curricular challenges within the CMG course. Structured modularization, curriculum alignment, and feedback-informed revisions were associated with enhanced learning and performance of students. These findings emphasize the value of continuous course/program evaluation for academic rigor and student engagement in graduate education.

## INTRODUCTION

Graduate programs lay the foundation of learning, development of skill and upgrading of professional values.^1^ They enhance employability of graduates and ensure long term career progression.^1^ These programs aligned with global and regional needs help develop skills and knowledge which improve job prospects and open opportunities in academia, research and industry.^2^ Master of Philosophy (MPhil) in biological and biomedical sciences in this regard, plays a crucial role in building applicable research skills thus improvising the long-term impact on healthcare particularly in low- and middle-income countries like Pakistan.^3^,^4^

As important as these graduate programs are, their evaluation and curricular reformation are equally critical to maintain effectiveness, relevance and evolving global needs. Literature suggests a burning need to evaluate the curriculum to ensure desired outcomes have been achieved.^5^ Evaluation is the process of gathering and analyzing data to assess the performance and effectiveness of a program, support evidence-based practice, and make informed decisions for program/course improvement.^6^,^7^

The Aga Khan University (AKU), a private institution based in Karachi, Pakistan, started a new graduate program, titled MPhil in Biological and Biomedical Sciences (BBS) in 2020. The goal of this program is to identify and nurture graduate students with talent and passion for research and learning in biological and biomedical sciences.^2^ The 2-3 years program, which nurtures students of diverse educational background, includes coursework in the first year and research-based thesis work in the second/third year. The core courses offered in the first year include: 1) Cellular, Molecular and Genetics Sciences: Knowledge and Application (CMG), 2) Research Methodology, 3) Biostatistics and Data Visualization in R, and 4) Research Seminars in Health Sciences. Discipline-specific elective courses are offered to students in the second semester of their coursework; these are aligned with their area of research interest and specialization.

For all the courses, sustained curriculum renewal and innovation is promoted to maintain a culture of quality and academic rigor. It communicates to both learners and other stakeholders, including external assessors and accreditors, that the program is agile, committed to remaining relevant, and meeting the desired competencies. It also provides opportunities for continuous professional development by encouraging faculty members to reflect on the effectiveness of the curricular contents and pedagogical approaches and respond by enhancing their own repertoire of teaching and learning approaches and innovating in their professional practice.^8^

The MPhil program in BBS has now graduated five cohorts and is positioned to be evaluated as part of continuous program monitoring and evaluation. The program aims to periodically evaluate all the courses, to improve the course’s alignment with required competencies, enhance student engagement and attainment of learning outcomes. Various course evaluation models are applied, and periodic course retreats are organized to support curriculum enhancement and continuous improvement. These include student feedback, faculty evaluations, course reviews, alumni input, committee discussions, and assessment analysis. Here we share the results of this evaluation and specifically, iterative modifications in CMG, course, as an exemplar, which was designed to elucidate the relationships between cell biology, molecular biology and molecular genetics by connecting dots of how cell functions are driven by molecules and are being controlled by genes. There is limited published evidence on framework-guided evaluation and outcome reporting of curricular reforms in integrated Cellular, Molecular and Genetic Sciences courses within graduate biomedical science programs, particularly in low- and middle-income country settings. The program office systematically collected feedback from students across successive cohorts of MPhil in BBS. End-of-year feedback in 2022 revealed that the course was cognitively demanding, with an excessive information load and a substantial assessment burden. Students also reported that assessments were neither optimally aligned with learning objectives nor appropriately scheduled. In response, the program committee undertook a comprehensive evaluation of the CMG course delivered in 2023 and subsequently in 2024, implemented evidence-informed curricular reforms, and evaluated the outcomes of the revised course offerings in 2024 and 2025. This study was designed to critically evaluate the CMG course, propose evidence-based reforms, compare student performance across cohorts before and after implementation of the reforms, and analyze student feedback to inform further course improvement.

## METHODOLOGY

A mixed-methods, CIPP-guided course evaluation of the CMG course was conducted between March 2024 and June 2025 The design integrated qualitative sources (document review; verbal student feedback during scheduled mid and end-of-module meetings; VLE-based structured written evaluations; and faculty reflections from course retreats) with quantitative data (student performance across the MPhil in BBS 2023, 2024, and 2025 cohorts).

### Ethical Approval:

(ERC approval: 2024-2211-28473; dated March 13, 2024).

### CIPP model:

Using the CIPP model,^9^ data was mapped to Context, Input, Process and Product domains as follows:

- ***Context:*** Program goals, student characteristics, data from prior implementation and delivery of CMG course.
- ***Input:*** Course outline, sequencing of content, Table of Specifications and assessment plan, and instructional resources.
- ***Process:*** Session delivery, timing, instructor coordination, and implementation of formative assessments.
- ***Product:*** Indicators of student performance/assessment results.

### Inclusion and exclusion criteria:

For qualitative components, all enrolled students were expected to provide written feedback on the VLE and invited to attend scheduled feedback meetings; the course and program faculty were included, contributing their reflections. For quantitative analyses, the performance of all students with complete CMG summative assessment records for the relevant academic year were included.

### Data collection:

Course documents (course outline, schedule, learning outcomes, assessment blueprint) were compiled. Mid- and end-of-module feedback meetings were facilitated by the course faculty leads using open-ended questions that align with CIPP domains; detailed minutes were recorded. Students submitted written module evaluations on the VLE. To ensure anonymity and confidentiality, all data sources were de-identified prior to analysis and stored on secure institutional drives.

### Data analysis:

Qualitative data sources were analyzed and mapped to the four CIPP domains. Coding and themes were reviewed by the study team and finalized after consensus. Trustworthiness of qualitative data was supported through triangulation across data sources (documents, student feedback, faculty reflections), maintenance of an audit trail (meeting minutes and approvals), and peer debriefing with the program office. Quantitative data (course grades) were summarized using descriptive statistics, specifically frequencies for grade categories. No inferential hypothesis testing was performed. Qualitative and quantitative findings were integrated at the interpretation stage through triangulation to identify convergent and divergent evidence, which informed iterative course refinements in 2024 and 2025.

## RESULTS

The number of students who attended the CMG course were 11, 12 and 16 in year 2023, 2024 and 2025 respectively. The data from desk records (schedules of course, learning objectives, assessment methods) and feedback (mid-module and end-of-module) from students and faculty was effectively incorporated in revising the curriculum (improved content sequencing, simplification of learning objectives and integration of formative assessments) is given in Table-I.

**Table-I:** Review and Reforms of the CMG Course in MPhil in BBS Program.

Reforms in CMG Course Offered in 2024				
Issue Identified in 2023	Source of Feedback	Intervention Implemented	Details of Change	Outcome
Poor sequencing of instructional content	Desk review of prior academic sessions	Comprehensive curricular realignment	CMG course divided into 3 modular segments: • Cellular Biology (5 weeks) • Molecular Biology (7 weeks) • Genetics (4 weeks) Table of Specification (ToS) created with learning objectives, strategies, assessments, and weightage	Improved conceptual flow and coherence across sessions, especially in multi-instructor format
Lack of practice materials for summative assessments	Desk review and informal student and faculty feedback	Emphasis on formative assessments and VLE integration	Targeted practice exercises (MCQs & SAQs) added to Molecular Biology module via VLE; Clear communication of assessment weightage	Increased student preparedness and timely feedback on performance
***Iterations in CMG Course Offered in 2025***				
*Issue Identified in 2024*	*Source of Feedback*	*Intervention Implemented*	*Details of Change*	*Outcome*
Genetics content perceived as overwhelming	Student post-course evaluations and faculty consensus	Simplification and eventual separation of genetics content	Immediate: Genetics retained in CMG with simplified focus on core principles	Enhanced engagement and understanding for students with limited prior exposure to genetics

The letter Grades in Table II are based on Score: A- = 85-89, B+=80-84, B = 75-79, B- = 70-74, C+ =65-69, C = 60-64. An ‘Incomplete’ grade was assigned to student who was unable to appear in the examination due to health-related issues Table III summarizes the distribution of student grades over three consecutive years (2023–2025) for the CMG course. It shows the number of students and the corresponding percentage achieving each letter grade, along with the associated grade point. The data highlights trend in academic performance from 2023-2025: the proportion of higher grades (A− and B+) has increased from 2023 to 2025, while the proportion of lower grades (C+, C) has decreased.

**Table-II:** Description of Letter Grade.

Letter Grade	Percentage Equivalent	Grade Point
A+	95–100	4.0
A	90–94	4.0
A−	85–89	3.7
B+	80–84	3.3
B	75–79	3.0
B−	70–74	2.7
C+	65–69	2.3
C	60–64	2.0
C−	55–59	1.7*
F	< 55	0.0
P	Pass	‡
I	Incomplete	‡

**Table-III:** Comparison of Grades in CMG course from 2023 to 2025.

Letter Grade	Grade Point	2023 (n=11)	2024 (n=12)	2025 (n=16)
A (90–94)	4.0	0 (0%)	0 (0%)	1 (6.25%)
A− (85–89)	3.7	0 (0%)	3 (25%)	4 (25%)
B+ (80–84)	3.3	0 (0%)	2 (16.7%)	4 (25%)
B (75–79)	3.0	1 (9%)	1 (8.3%)	1 (6.25%)
B− (70–74)	2.7	5 (45%)	6 (50%)	4 (25%)
C+ (65–69)	2.3	4 (36%)	2 (16.7%)	0 (0%)
C (60–64)	2.0	2 (18%)	1 (8.3%)	0 (0%)
C− (55–59)	1.7	0 (0%)	0 (0%)	1 (6.25%)
Incomplete (I)	—	0	0	1

## DISCUSSION

The iterative curricular revisions in our study for the core MPhil course of CMG based on institutional records and stakeholder feedback, demonstrably enhanced student academic performance across two annual cycles. Initial reforms in 2024 yielded improved overall grades, a trend which amplified significantly in the 2025 iteration with a pronounced shift toward higher-grade brackets (A- and B+) and elimination of lower-tier grades (C+ and C), showing sustained efficacy of the targeted interventions in content sequencing, learning objectives, and assessment strategy. The reforms reflect dynamic and continuously evolving curriculum in response to diverse stakeholder needs and transformation after incorporation of feedback from the students.^10^ Similar studies supports regular monitoring, systematic evaluation, and proactive enhancement to maintain effectiveness of the curriculum.^11^,^12^ In addition to that curricular reforms can enhance students’ performance by improving curriculum quality, teaching method and students’ participation.^13^

Global literature has emphasized the need for graduate programs to be routinely revised to align with scientific advances and workforce demands.^14^,^15^ A landmark report by Zhang Nan in 2025 highlighted the need for transformative, adaptable curricula particularly in health-related educational programs to meet global health challenges.^16^ Within Pakistan, the discourse on curriculum reform has largely centered on undergraduate medical education. The national Undergraduate Medical Education (UGME) curriculum, last comprehensively updated in 2005 and revised in 2022, has been examined through the perspectives of academic leaders, who expressed broad support for reform while identifying implementation barriers such as limited trained faculty and financial constraints.^17^ These discussions, however, remain predominantly focused on undergraduate training. In contrast, there is comparatively limited published evidence addressing curricular reform at the graduate level in biomedical sciences. Our focus on restructuring the CMG course responds to this gap and aligns with the growing need for continuous curriculum renewal in graduate biomedical and health sciences education. In Pakistan, postgraduate training in basic and biomedical sciences has increasingly emphasized structured learning outcomes and research competence, yet systemic challenges, including faculty development needs and resource limitations, remain significant. Editorial perspectives on level-IV postgraduate education in basic sciences underscore the importance of strengthening graduate programs to enhance educational quality, while highlighting gaps in advanced competency integration.^18^ These national insights reinforce the imperative for competency-aligned and institutionally supported curricular frameworks at the graduate level, priorities that are reflected in the modular and adaptive redesign of the CMG course.

The experience of the MPhil in BBS at AKU described here highlights the value of evidence-informed stakeholder-informed curricular revision. Continuous curricular renewal also ensures that a program remains responsive to changes in knowledge base, advances in the field and societal needs.

The restructured, modular redesign of the CMG course which separates Cell Biology, Molecular Biology, and Genetics addressed the critical need for curricular sequencing. This issue had come up strongly during collection of feedback from students and faculty. In disciplines characterized by complex interrelated concepts, such as molecular and cellular sciences, unstructured integration of content can overwhelm working memory and impede meaningful learning.^19^ This aligns with principles derived from Cognitive Load Theory which proposes that working memory has limited capacity and that instructional designs must promote the development of organized knowledge structures and long-term retention.^20^ By sequencing foundational concepts and distributing related content into coherent modules, the revised CMG curriculum likely reduced extraneous cognitive load while optimizing intrinsic load, thereby supporting deeper conceptual integration which resulted in better performance. The inclusion of a modified and detailed Table of Specification (ToS) ensured intentional alignment between objectives, teaching methods, and assessments, reflecting best practices in curriculum design.^21^ The challenges students faced with the genetic component of CMG particularly due to its complexity amid dense, integrated content were addressed by our approach of simplifying genetics content in CMG course and shifting advanced genetic content into a separate course.^22^

The integration of targeted formative assessments in CMG also proved to be helpful in ensuring improved student performance for the exams. This initiative is strongly supported by evidence demonstrating that formative assessments enhance summative exam performance and reduces exam anxiety.^23^ Overall, the restructuring of CMG course reflects a thoughtful and responsive approach to curriculum design, highlighting how modular sequencing can improve conceptual flow and clarity, ease cognitive load, and make complex scientific content more accessible for diverse learners to ensure its continued relevance and alignment with desired learning outcomes.

Looking ahead, we plan to deliver detailed genetics content as part of another elective course titled Genetics, Genomics and Bioinformatics, from the next academic year. The integrated CMG core course will remain as four-credit hours, giving more curricular time to Cell and molecular biology concepts. This approach will allow more time for in-depth exploration of core concepts, thereby promoting better comprehension and retention. The program will also continue to assess its effectiveness and incorporate curricular and programmatic revisions as needed, for example in areas related to artificial intelligence.^24^

### Limitations:

This study is not without limitations. The comparison across different student cohorts introduces potential cohort effects and unmeasured confounding factors that may have influenced outcomes independent of the curricular redesign. Second, although assessment formats were aligned, exam non-equivalence or difficulty drift cannot be ruled out. Variations in instructor involvement and teaching approaches across modules may also have contributed to differences in learning outcomes. In addition, student feedback data may be subject to response bias. Finally, as a single-centered study, the findings may not be generalizable. Moreover, students’ satisfaction after implementation of reforms was not assessed, and being a single-centered study results could not be generalized. Future evaluations should incorporate this data to provide a more comprehensive understanding of the outcomes of the reform.

## CONCLUSION

The structured evaluation and targeted reform of the CMG course led to measurable improvements in student performance and learning experience. Findings from student feedback, faculty insights, and academic outcomes demonstrate that modular restructuring, simplified content delivery, and enhanced formative assessment strategies significantly enhanced the coherence, accessibility, and effectiveness of the course. These results emphasize the value of continuous curriculum review and evidence-based modifications in fostering academic success in graduate biomedical education.

## References

[1] Maghamil C (2025). Graduate Education and Its Influence on Employability and Career Progression: A Tracer Study of La Salle University Graduates. Asian J Educ.

[2] Rehman R, Ali R, Khan R, Arain FM (2017). An organizational challenge: reframing of leadership to introduce 'Master's program in Basic Science'. J Bahria Uni Med Dent Coll.

[3] Cordrey T, Thomas A, King E, Gustafson O (2024). Evaluating the perceived impact and legacy of master's degree level research in the allied health professions: a UK-wide cross-sectional survey. BMC Med Educ.

[4] Holloway S, Taylor A, Tombs M (2020). Impact of postgraduate study on healthcare professionals'academic and clinical practice. Br. J Health Care Manag.

[5] Onyura B, Lass E, Lazor J, Zuccaro L, Hamza DM (2022). Vitalizing the evaluation of curricular implementation: a framework for attending to the “how and whys”of curriculum evolution. Adv Health Sci Educ.

[6] Sobodić A, Balaban I, Granić A (2024). The impact of usability factors on continuance intention to use the system for acquisition and evaluation of digital competences in the domain of education. Technol Soc.

[7] Gerlach B, Faulkner M, Ding X, Franklin C (2024). Evaluating intervention and program effectiveness. The school services sourcebook: A guide for school-based professionals.

[8] Toshmatov BE, Abdumajidov Sh F (2024). Improving the teaching profession. Int J Pedagogics.

[9] Stufflebeam DL, Coryn CL (2014). Evaluation theory, models, and applications. John Wiley &Sons.

[10] Aslam K, Khan RA, Aslam MA, Zaidi FZ (2024). Curriculum implementation challenges: Development and validation of an integrated curriculum implementation challenges tool. Pak J Med Sci.

[11] Kern DE, Thomas PA, Hughes MT, Chen BY (2016). Curriculum development for medical education: A six-step approach.

[12] Dent JA, Harden RM, &Hunt D (2021). A Practical Guide for Medical Teachers (6th ed.).

[13] Sosa MA, Garg N, Onge JS, Issenberg B, Diaz Y (2024). A graduate medical education (GME) quality improvement curriculum leads to improved knowledge and participation in high quality improvement projects by trainees. Int J Med Inform.

[14] National Academies of Sciences, Engineering and Medicine (2018). Graduate STEM Education for the 21st Century.

[15] El-Hashash A (2024). Reforming Biological/Biomedical Science Teaching and Education: A Reflection on Well-Developed Practices and Approaches in Large Class. Int J Cybern Inf.

[16] Nan Z (2025). Transforming Nursing Education: A Conceptual Overview of Curriculum Redesign Models. Curriculum Teach Methodol.

[17] Bakhshi SK, Afzal N, Merchant AAH, Abdul Rahim K, Shaikh NQ, Noorali AA (2024). Undergraduate Medical Education Curriculum Reforms in Pakistan: A Mixed Methods Study of Academic Leadership Perspectives. Acad Med.

[18] Zahid P (2025). Role of Level-IV Post-graduation in Basic Sciences in Research Development and Quality Improvement of Graduate Medical Education in Pakistan. J Fatima Jinnah Med Univ.

[19] van Merriënboer JJ, Sweller J (2010). Cognitive load theory in health professional education: design principles and strategies. Med Educ.

[20] Skulmowski A, Xu KM (2022). Understanding cognitive load in digital and online learning: A new perspective on extraneous cognitive load. Educ. Psychol Rev.

[21] Dasgupta S, Symes K, Hyman L (2015). Leading change: Curriculum reform in graduate education in the biomedical sciences. Biochem Mol Biol Educ.

[22] Kapur S, Lichten L, Ali N, Garber KB (2024). Poor recall of genetics curriculum by medical students highlights barriers to use in clinical practice. J Genet Couns.

[23] Beaton LL (2025). Online Activities as Formative Assessment-a Comparison of two Methods. J Educ Technol Syst.

[24] Rincon J, Pelletier AR, Gilliland D, Wang W, Wang D, Sankar BS (2505). Bridge2AI: Building a cross-disciplinary curriculum towards AI-enhanced biomedical and clinical care. 2025.arXiv preprint arXiv.

